# Stimuli-responsive biomaterials for cardiac tissue engineering and dynamic mechanobiology

**DOI:** 10.1063/5.0025378

**Published:** 2021-03-02

**Authors:** Huaiyu Shi, Chenyan Wang, Zhen Ma

**Affiliations:** 1 Department of Biomedical and Chemical Engineering, Syracuse University, Syracuse, New York 13244, USA; 2 BioInspired Syracuse Institute for Material and Living Systems, Syracuse University, Syracuse, New York 13244, USA

## Abstract

Since the term “smart materials” was put forward in the 1980s, stimuli-responsive biomaterials have been used as powerful tools in tissue engineering, mechanobiology, and clinical applications. For the purpose of myocardial repair and regeneration, stimuli-responsive biomaterials are employed to fabricate hydrogels and nanoparticles for targeted delivery of therapeutic drugs and cells, which have been proved to alleviate disease progression and enhance tissue regeneration. By reproducing the sophisticated and dynamic microenvironment of the native heart, stimuli-responsive biomaterials have also been used to engineer dynamic culture systems to understand how cardiac cells and tissues respond to progressive changes in extracellular microenvironments, enabling the investigation of dynamic cell mechanobiology. Here, we provide an overview of stimuli-responsive biomaterials used in cardiovascular research applications, with a specific focus on cardiac tissue engineering and dynamic cell mechanobiology. We also discuss how these smart materials can be utilized to mimic the dynamic microenvironment during heart development, which might provide an opportunity to reveal the fundamental mechanisms of cardiomyogenesis and cardiac maturation.

## I. INTRODUCTION

Human myocardium is constantly regulated by dynamic external microenvironmental cues, including biochemical, mechanical, and electrical signals. These signals propagate through cell–cell or cell–extracellular matrix (ECM) interfaces and act on intracellular signaling pathways. The order of electrical signal transmission across the cardiomyocytes is essential for maintaining the rhythmic contraction–relaxation cycle of myocardium and unidirectional blood flow at adequate pressure.^1^ Moreover, cardiomyocytes constantly experience mechanical stretching from contracting myocardium and shear stress from blood flow. For example, cardiac hypertrophy is a cardiac remodeling process that compensates for cardiac overload. It has been found that melusin, a muscle-specific integrin-binding protein, contributes to hypertrophic responses by transducing mechanical signals to intracellular pathways.^2^ In addition to cardiomyocytes, mechanical signals also play a significant role in the pathophysiological phenotype transformation of other cell types within the myocardium. For instance, cardiac fibroblasts are differentiated from epicardial cells during embryonic development through the activation of the Hippo–YAP pathway, which is triggered by mechanical tension.^3,4^ Furthermore, mechanical stress induced by hypertrophic remodeling or other injuries has been shown to mediate the activation of myofibroblasts.^5^

In striving to mimic the native condition of myocardium *in vitro*, researchers have pursued many different approaches to create a dynamic environment by incorporating external stimulations or active culture systems.^6–12^ In particular, in recent advances in work on cardiomyocytes derived from human induced pluripotent stem cells (hiPSC-CMs), active electrical and mechanical stimulations have been proved to promote hiPSC-CM differentiation and maturation, manifested through upregulated expression of cardiac markers, acceleration of Ca^2+^ cycles, and enhancement of contractile function.^7,8^ Using a rocker, a dynamic culture platform was established to promote cell survival and maturation of cardiomyocytes.^13^ Recently, a comparison was made for cardiomyocyte alignment between structural cues from aligned nanofibrous scaffolds and electrical stimulation from a unidirectional electrical field. Interestingly, electrical stimulation showed higher efficiency in the improvement of hiPSC-CM alignment and the expression of cardiac-specific markers.^14^ However, these fast-changing external interventions could not reproduce the gradual changes in and spatial heterogeneity of the cardiac microenvironment during heart development and remodeling. For *in vivo* studies, these invasive techniques are not feasible for further manipulation of cells after transplantation. Therefore, researchers have dedicated their effort to incorporating dynamic properties in biological scaffolds, using stimuli-responsive biomaterials to achieve spatiotemporal control of the cellular microenvironment.

Stimuli-responsive materials, which undergo property switches in response to specific external stimuli, are able to provide an on-demand temporal change in environmental signals to cultured cells. Barium titanate, one of the first stimuli-responsive materials, is an inorganic compound exhibiting the piezoelectric effect.^15^ After decades of endeavor, the class of stimuli-responsive materials has been broadened tremendously. Based on the category of stimuli, these smart materials can be classified as thermo-responsive,^16^ photo-responsive,^17^ pH-responsive,^18^ mechanical-responsive,^19^ potential-responsive,^20^ magneto-responsive,^21^ and biochemical-responsive polymers.^22^ The excellent programmability of stimuli-responsive materials makes them promising candidates for providing dynamic biochemical and biomechanical cues to cells and tissues. For instance, polydimethylsiloxane (PDMS)-based micro-post arrays with incorporated cobalt nanowires can respond to a magnetic field and generate spatial movement to apply external forces to cells.^23^ In another example, thermo-responsive poly(*N*-isopropylacrylamide) (PNIPAAm) hydrogel was used to provide compressive or expansive tension to cells based on hydrogel swelling or shrinking at different temperatures.^24^ Additionally, the PNIPAAm hydrogel also showed tunable elasticity, depending on the environmental temperature.^25^ A photo-responsive polymer, azobenzene, demonstrated reversible *trans*-to-*cis* isomerization at different wavelengths of light. With the incorporation of azobenzene, the elasticity of poly(ethylene glycol) (PEG) hydrogel could be reversibly modulated by switching the light exposure between visible and ultraviolet (UV) light and could be used to provide dynamic biophysical controls to the encapsulated cells.^26^ Using nucleic acid sequences as crosslinkers, biopolymers were fabricated with tunable properties that could be controlled based on the complementary hybridization of different nucleotides. By copolymerizing monomers with designed nucleic acid crosslinkers, the stiffness of the DNA-containing hydrogel substrate could be modulated by adding exogenous DNA sequences that were complementary to the crosslinkers in the hydrogel.^27,28^ Using this dynamic system, both fibroblasts and neurons exhibited significant shape changes, induced by this stiffening process.

**FIG. 1.**
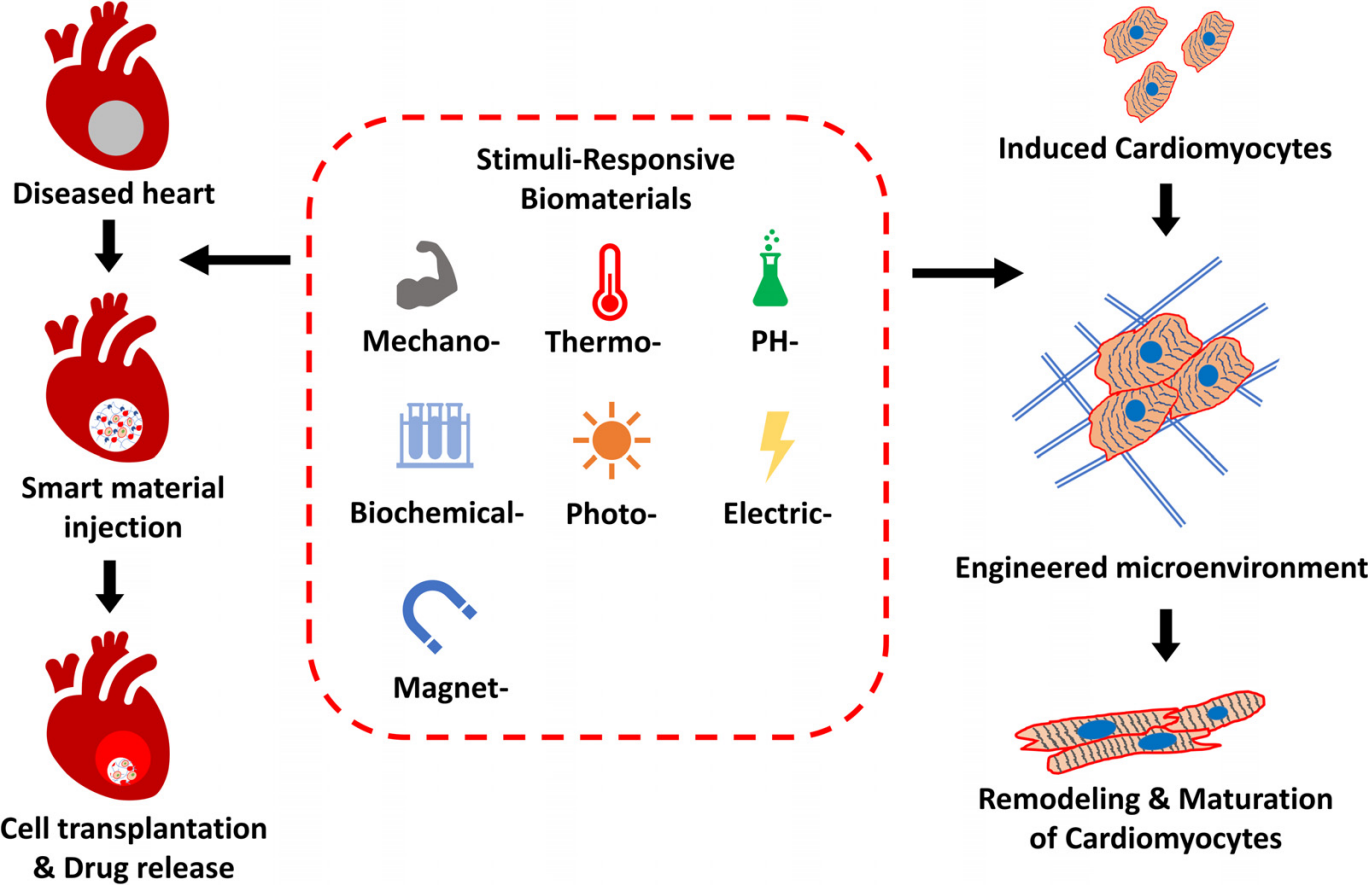
Applications of stimuli-responsive biomaterials in cardiac research. Stimuli-responsive biomaterials have been widely used for the application of targeted delivery of therapeutic drugs and cells for myocardial repair and regeneration. Stimuli-responsive biomaterials have also been the subject of increasing interest in the study of cardiac dynamic mechanobiology to promote the maturation of cardiomyocytes.

Specifically in the field of cardiac tissue engineering, stimuli-responsive materials have been used to endow synthetic ECMs with the ability to change their material properties dynamically, mimicking the natural scenarios of heart development with progressive changes in the chemical and physical microenvironment. Furthermore, stimuli-responsive materials could be engineered with cardiac-specific or even cardiovascular disease-specific responsiveness, to enhance the targeted delivery of therapeutic components to desired regions. In this article, we discuss different cardiac pathophysiological stimuli and different types of smart biomaterial for cell transplantation and drug delivery application, with the aim of tissue repair and regeneration. Next, we highlight the recent development of stimuli-responsive biomaterials for creating dynamic cell microenvironments for cardiac mechanobiological studies (Fig. 1).

## II. STIMULI-RESPONSIVE BIOMATERIALS IN CARDIAC TISSUE REGENERATION

Heart regeneration is widely acknowledged as a challenging subject, owing to the terminated stage and low turnover rate of cardiomyocytes after their maturation. To endow diseased hearts with renewability, drug delivery and cell transplantation have been extensively investigated to activate resident myocardial progenitor cells and supply regenerative components around damaged tissues. There have been successful demonstrations of many different types of carriers for *in vivo* delivery systems that could stabilize the nature of drugs and improve drug loading capacity.^29–32^ However, most of these systems are still impaired by a high risk of premature release in the circulation system and a lack of precise management of release profile at the target site.^33^ These disadvantages are the main reasons for undesired distribution of therapeutic agents and decreased clinical outcomes. Making use of on-demand property switching of stimuli-responsive materials, smart delivery systems are being developed to enable the disease-specific release of encapsulated components based on an understanding of pathological conditions.^34,35^ This could further enhance therapeutic efficiency by optimizing the spatiotemporal control of the payload concentration.^33^ In cardiac pathology, many disease-relevant environmental stimuli, including temperature, pH, enzymatic activities, and redox condition, have been applied to the design of smart systems for cell transplantation and drug delivery to facilitate heart repair.^36–39^ From bulk hydrogels to supramolecular hydrogels and nanoparticles, they could be programed with the same stimulus responsiveness but vary in drug cargos and releasing mechanisms.

### A. Smart hydrogels for cell transplantation

Smart hydrogels are advanced types of hydrogel that could undergo a dramatic and reversible volume phase change or sol–gel transition in response to a slight change in an environmental stimulus.^40^ They are widely used as carriers for both cell transplantation and drug delivery, owing to their great compatibility with various therapeutic agents, linked through either physical encapsulation or chemical bonding. Their fast gelation protects cells and drugs from harsh microenvironments within the native myocardium, and their soft-tissue-like properties help to maintain the nature of incorporated components.^41,42^ In addition to smart hydrogels, supramolecular hydrogels, which are assembled by pure noncovalent interactions between hydrogel molecules, are new carriers for delivering therapeutic agents. The flexibility from their dynamic building blocks allows for more customizable sol–gel transition and release kinetics.^43^

**FIG. 2.**
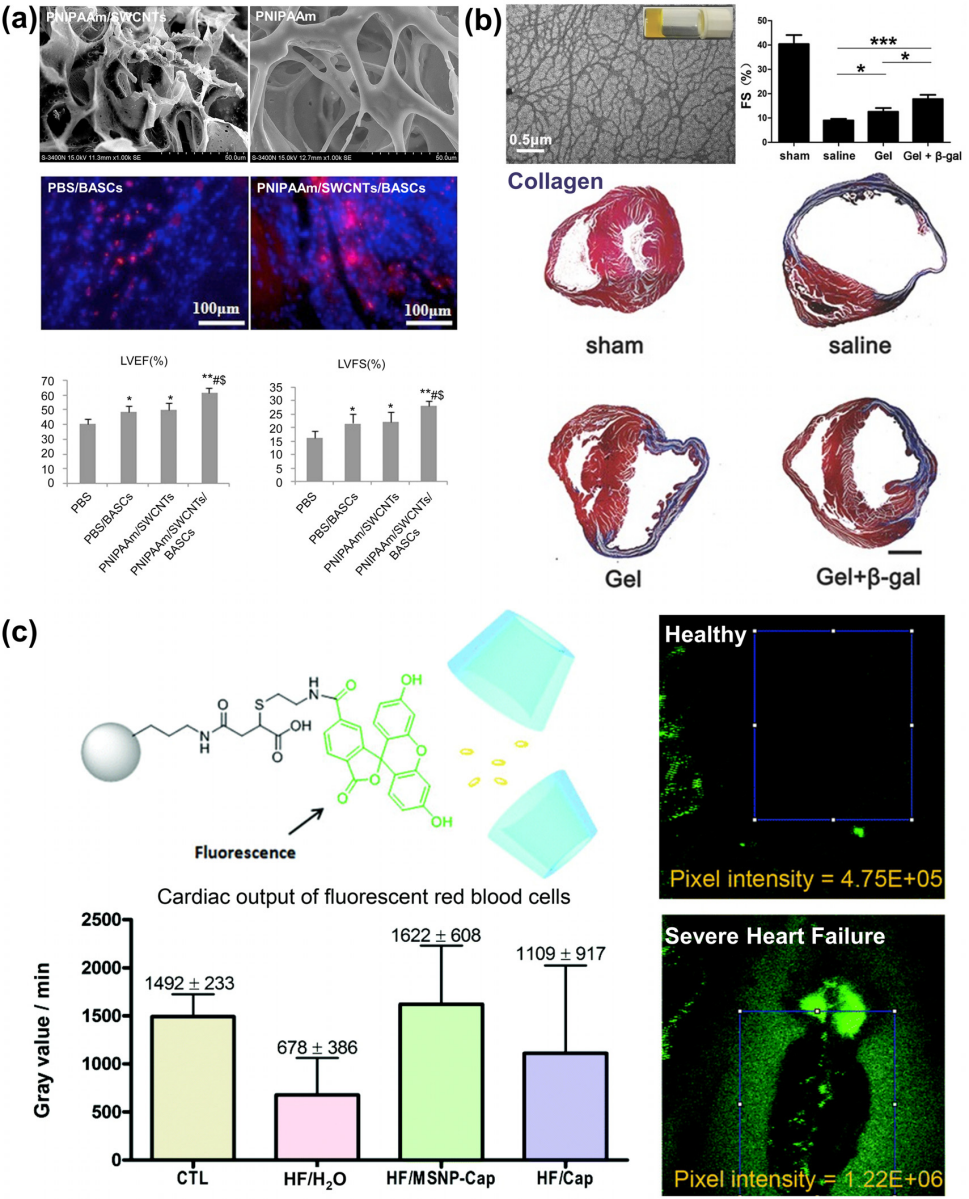
Stimuli-responsive biomaterials for cardiac tissue regeneration. (a) Single-wall carbon nanotubes (SWCNTs) were dispersed in PNIPAAm smart hydrogels to enhance the adhesion of ADSCs. Fluorescence images showed more ADSCs (red) in the transplantation area. As a result, heart contractile functions (LVEF and LVFS) were improved.^45^ Reprinted with permission from Li *et al.*, “A PNIPAAm-based thermosensitive hydrogel containing SWCNTs for stem cell transplantation in myocardial repair,” Biomaterials **35**(22), 5679–5688 (2014). Copyright 2014 Elsevier. (b) The dual release of NO and curcumin from β-galactosidase-responsive supramolecular hydrogels could decrease the collagen content, as indicated by Masson's trichrome staining. The fraction shortening for the mice with acute MI was also improved.^56^ Reprinted with permission from Chen *et al.*, “A mixed component supramolecular hydrogel to improve mice cardiac function and alleviate ventricular remodeling after acute myocardial infarction,” Adv. Funct. Mater. **27**(34), 1701798 (2017). Copyright 2017 Wiley. (c) H_2_O_2_-responsive fluorescent probes and captopril were loaded in nanoparticles, which could simultaneously evaluate the severity of heart failure based on the probe fluorescence intensity and enhance the cardiac outputs in a zebrafish model.^61^ Republished with permission from Tan *et al.*, “Responsive mesoporous silica nanoparticles for sensing of hydrogen peroxide and simultaneous treatment toward heart failure,” Nanoscale **9**(6), 2253 (2017).Copyright 2017 Royal Society of Chemistry.

The PNIPAAm hydrogels have outstanding mobility below 32 °C and fast *in situ* gelation after injection, making them a popular option for cell transplantation.^44^ However, their bioactivity is not sufficient to support the adhesion and proliferation of encapsulated cells. To enhance cell–material interactions in PNIPAAm hydrogels, single-wall carbon nanotubes (SWCNTs) were dispersed uniformly in a hydrogel solution.^45^ These SWCNT-modified hydrogels maintained a similar gelation profile to pure PNIPAAm but showed a significant increase in surface roughness, which led to better adhesion and spreading of adipose-derived stem cells (ADSCs). One week after intra-abdominal injection in rat models, the number of ADSCs in a group injected with PNIPAAm, SWCNTs, and ADSCs was much higher than that in a control group injected with phosphate-buffered saline and ADSCs, indicating the improvement of ADSC retention in the host myocardium. Significant improvements in left ventricle ejection fraction (LVEF) and left ventricle fraction shortening (LVFS) were accompanied by the decreased infarct size and increased wall thickness [Fig. 2(a)].

Furthermore, PNIPAAm could be combined with other smart hydrogels to generate dual-responsive hydrogels. Copolymerization of *N*-isopropylacrylamide (NIPAAm), polyacrylamide (PAA), methacrylate poly(ethylene glycol) methyl ether (MA-PEG), and the macromer 2-hydroxyethyl methacrylate-co-oligo (trimethylene carbonate) (HEMA-oTMC) was used to synthesize a family of hydrogels that were sensitive to both pH and temperature variation;^38^ NIPAAm and PAA were used to introduce thermal and pH sensitivity, while HEMA-oTMC and MA-PEG were used to make additional adjustments to the critical gelation temperature. This complex hydrogel could solidify in an infarcted heart, with a pH of 6–7, but liquefy in the bloodstream, with a pH of 7.4. Although no animal model was used for cell and hydrogel transplantation in this study, cardiosphere-derived cells (CDCs) were encapsulated in this hydrogel and showed strong viability after one week of culture. The CDCs also exhibited differentiation toward the cardiac lineage, evidenced by the expression of cardiac markers such as troponin T, myosin heavy chain α, and calcium ion channel proteins.

Chitosan is a biocompatible and biodegradable polymer that is often used to fabricate scaffolds for tissue engineering applications. Using glycerol phosphate (GP) as an ionic cross-linking agent, chitosan could be made into a temperature-sensitive smart hydrogel.^46^ Anionic GPs are able to bind with positive-charged chitosan chains, and their hydroxy groups trigger the formation of hydrogen bonds with water. In this way, GPs could physically separate the chitosan polymer chains by building a protective layer around them. When the temperature increases, disruption of the protective layer leads to hydrophobic bonding between chitosan chains to induce the gelation process.^47^ Using this chitosan–GP system, embryonic stem cells (ESCs) were transplanted into an ischemic rat model for the purpose of heart regeneration.^48^ Compared with cell-only control groups, there was a significant increase in the number of cells and the graft size in the hydrogel-encapsulated groups. Heart functions, wall thickness, and micro-vessel density were all improved after 4 weeks, as chitosan degraded gradually to release the ESCs. Like most hydrogels, chitosan–GP gels have low mechanical strength; thus, they might not tolerate the high mechanical stress in native heart tissues. To solve this problem, gold nanoparticles (GNPs) were dispersed in the chitosan–GP gels to fabricate a composite that resembled the electromechanical properties of the myocardium without compromising on thermosensitivity.^49^ Mesenchymal stem cells (MSCs) seeded on the chitosan–GP–GNP hydrogels showed enhanced cardiomyogenic differentiation, compared with cells on pure chitosan hydrogels.

### B. Smart hydrogels for drug delivery

In addition to cell transplantation, stimuli-responsive polymers are also used for therapeutic drug delivery for cardiac therapy. Two triblock polymers were combined to fabricate a flower-type micelle structure with dual therapeutic functions.^50^ One triblock polymer contained l-arginine, which could be converted into nitric oxide (NO), mediated by the accumulated macrophages at the injury site. The other triblock polymer proved to be capable of scavenging reactive oxygen species (ROS) and alleviating tissue injury. Unlike conventional temperature-responsive hydrogels, the gelation of this system was enabled by irreversible electrostatic cross-linking between micelles. When the temperature increased, a partial disintegration of the flower core resulting from elevated ionic strength led to the gradual release of functional components. Results from myocardial infarction (MI) mouse models showed that this composite solidified immediately after intracardiac injection and was homogeneously distributed at the injected site for more than 10 days. Treated groups showed obvious restoration of the wall thickness, improved cardiac functions (LVEF and LVFS), decreased infarction size, and pro-angiogenesis near the injection sites.

In addition to hydrogels responding to a single stimulus, dual-responsive hydrogels with enhanced gelation restrictions have been developed for drug delivery. Based on copolymerization of NIPAAm, PAA, and butyl acrylate (BA), a polymer with responsiveness to both temperature and pH was fabricated to deliver basic fibroblast growth factors (bFGFs) to infarcted rat myocardium.^51^ To achieve targeted delivery of bFGFs to the acidic heart injury site, the polymer took on a liquid state at room temperature and pH 7.4 but transitioned to a solid state at 37 °C and pH 6.8. The bFGFs were simply incorporated into the polymer by gentle mixing at a predefined concentration. Owing to the local retention provided by the hydrogel, the concentration of bFGFs increased over time following injection at the apex of the heart, while there was little to no detection of bFGFs in the basal part of the heart. The release of bFGFs induced an increase in capillary and arteriolar densities, along with a twofold improvement of blood flow to the MI area.

Local enzymatic activities within a diseased heart usually exhibit a distinct profile compared with that of healthy cardiac tissue. A representative characteristic is the overexpression of matrix metalloproteinases (MMPs), which cleave ECM components, decrease the mechanical integrity of the myocardium, and cause deterioration of cardiac functions.^52,53^ Therefore, attenuation of abnormal upregulation of MMPs has become a popular clinical target in the treatment of various heart diseases. A recently developed approach to fabricating enzyme-responsive hydrogels was achieved based on the genetic fusion of peptide sequences with bFGFs,^37^ which were tagged with a glutathione *S*-transferase sequence that could specifically bind to the glutathione-modified collagen hydrogel, with an MMP-2/9-cleavable peptide, TIMP, enclosed between the bFGFs and the glutathione. When exposed to the infarcted heart, TIMP would serve as a competitive substrate for MMPs to inhibit their ability of ECM degradation. Meanwhile, cleavage by MMPs caused the dissociation of bFGFs from the hydrogel to initiate angiogenesis progress. Treated rat MI models showed alleviation of ventricular wall thinning, reduction of collagen deposition, and improvement of cardiac contractions. Hydrogels made of self-assembling peptides (SAPs) are attractive as injectable biomaterials, owing to their structure, which resemble the native ECM and are amenable to sequence modification. For example, a recognition sequence for MMPs or elastases was tagged onto cyclic peptide progelators.^54^ After a minimally invasive catheter injection, enzymatic cleavage of the progelators resulted in the linearization of cyclic peptides to produce SAPs, which then assembled to form a re-healable viscoelastic hydrogel.

A supramolecular hydrogel was developed by modifying PEG hydrogel with ureido-pyrimidinone units.^55^ This hydrogel could remain in a liquid state at a basic pH and reversibly transition into a gel state at a neutral pH by breaking inter-fiber crosslinks that formed transient supramolecular networks. Hepatocyte growth factor, insulin-like growth factor-1, and MRI contrast agents were co-delivered to a porcine acute MI model. The controlled release of growth factors led to a decrease in the local collagen content and the generation of viable myocardium clusters. In another study, a β-galactosidase-responsive hydrogelator that could release NO was mixed with curcumin to form a supramolecular hydrogel mixture.^56^ Curcumin has anti-inflammatory, anti-oxidant, and anti-apoptotic properties, while NO contributes to native angiogenesis. The addition of β-galactosidase further enhanced the therapeutic efficacy of this system. Dual release of NO and curcumin improved the heart performance remarkably, as demonstrated by improved cardiac functions, reduced collagen deposition, suppressed cell apoptosis, and increased neovascularization [Fig. 2(b)].

### C. Nanoparticles and nanogels

Nanoparticle-mediated cell and drug delivery has received much attention, owing to the small size and high surface-to-volume ratio of nanoparticles. These advantages benefit noninvasive administration and improve drug loading capacity.^57,58^ Nanoparticles are highly compatible with various surface functionalizations, enabling control of pharmacokinetics and biodistribution.^59^ Therefore, nanoparticles have been largely used to develop smart vehicles for targeted drug delivery in response to specialized environmental stimuli.

An MMP-specific sequence was linked on a polynorbornene backbone to form nanoparticles with enzymatic sensitivity.^60^ The polymer amphiphilicity was optimized to increase the responsiveness by reducing the hydrophilic weight fraction. When MMPs were present, discrete micellar nanoparticles would undergo a morphological transition to form a network-like scaffold, which facilitated the aggregation of nanoparticles at the targeted area. To investigate the specificity of this system to the infarcted heart, responsive nanoparticles were injected in both healthy and MI rat models. The nanoparticles only accumulated at the infarction area and adjacent border zone, while no aggregates were observed in the healthy rats. The pattern of disease-specific nanoparticle accumulation continued for up to 28 days, indicating the promise of this responsive nanoparticle as a long-term drug release reservoir.

A landmark feature of MI is the overproduction of ROS, such as hydrogen peroxide (H_2_O_2_) and hydroxyl radicals. These ROS would damage cardiomyocytes, promote inflammation, and induce fibrosis. An H_2_O_2_-responsive nanoparticle made of polyoxalate was used to scavenge ROS and release vanillyl alcohol, which has anti-oxidant, anti-inflammatory, and anti-apoptotic properties.^36^ The H_2_O_2_ responsiveness was built on the introduction of peroxalate ester linkage, which could react with H_2_O_2_ for hydrolytic degradation of nanoparticles. The ability of nanoparticles injected in doxorubicin-induced cardiomyopathic mice to reduce oxidative stress and inflammatory responses was confirmed by the suppressed expression of inflammatory markers and NO synthases. In another work, boronic ester-based fluorescent probes were conjugated to nanoparticles in which a therapeutic drug, captopril, was loaded; the drug was protected by the bonds formed between α-cyclodextrin (α-CD) and H_2_O_2_ probes.^61^ When H_2_O_2_ was present, the fluorescent probes were “turned on” to induce the structural dissociation of α-CD, which then caused the release of captopril. This system offered a reliable way of diagnosing the severity of heart failure, which was proportional to the H_2_O_2_ concentration. Meanwhile, captopril delivery greatly improved the heartbeat rate and cardiac output [Fig. 2(c)].

Nanogels are made of polymer nanoparticles swelled in water; their 3D nanoscopic networks improve the response rate of smart hydrogels. Nanogels are more stable in the bloodstream than traditional hydrogels and could further facilitate cell retention in desired regions.^62,63^ In one study, PNIPAAm was made into nanogels using emulsion polymerization. Human cardiac stem cells (CSCs) were mixed with liquid nanogels at a ratio of 1:3, and gelation was triggered by increasing the temperature. The aggregation of nanogels surrounding the CSCs could encapsulate the cells for transplantation procedures. The porous and convoluted inner structure allowed for efficient diffusion of nutrients, oxygen, and regenerative factors from encapsulated CSCs.^64^ The CSCs promoted cardiac repair through the inhibition of apoptosis and induction of angiomyogenesis. Conversely, the capillary force resulted from small pore size-protected nanogel-encapsulated CSCs from immune cells, which did not elicit obvious immune rejection for both mouse and porcine models.

Although there have been plenty of successful demonstrations for the utilization of stimuli-responsive biomaterials to enhance treatment efficacy, current research effort is mainly devoted to controlling the release profile of therapeutic components. However, much less attention has been paid to evaluation of the post-delivery integrity of drugs or cells. In fact, during the property change of stimuli-responsive materials, there is a high risk of functional alterations of the encapsulated counterparts, especially for living cells, which are consistently regulated by their dynamic microenvironment. To better depict the cell–material interactions, *in vitro* dynamic models based on stimuli-responsive biomaterial scaffolds have gained much research interest in recent years. In particular, biomaterial scaffolds with tunable mechanical properties have played a significant role in the modulation of cardiac responses. The advancement of stimuli-responsive biomaterials has great potential to make a profound contribution to the field of cardiac mechanobiology, which can guide the future design for cardiac tissue regeneration applications.

## III. STIMULI-RESPONSIVE BIOMATERIALS IN DYNAMIC CARDIAC MECHANOBIOLOGY

In developmental and regenerative mechanobiology, it is crucial to understand how time-dependent biophysical cues affect cells during tissue formation and repair. Nearly a century of research in mechanobiology has implicated biophysical cues as critical regulators of cellular morphology, differentiation, and function in each sequential phase of tissue development or healing. However, current understanding and conceptual models are still based largely on results from studies of static experimental platforms in which biophysical cues remain constant over time. Recent effort in the field has pursued *in vitro* approaches that recapitulate time-dependent biophysical cues to enable the study of dynamic mechanobiology of different biological systems.

### A. Stimuli in cardiac mechanobiology

Pathological remodeling of the heart often leads to changes in ECM properties, such as stiffness, topography, or viscoelasticity, further affecting cardiac contractility. To mimic the dynamic changes of ECM properties, a variety of biomaterial substrates have been fabricated with different mechanical features. The spreading, shape, and gelatinase secretion of cardiomyocytes and cardiac fibroblasts cultured on PDMS-based substrates showed a positive relationship with the stiffness of substrates.^65^ Similarly, a polyacrylamide-based substrate with tunable stiffness was also used to control the adhesion properties and contractile functions of cardiomyocytes.^66,67^ Specifically, a soft substrate with a stiffness of 10 kPa was found to promote sarcomere organization and enhance the contractile force of single cardiomyocytes.^68–70^ The alignment and functionality of cardiomyocytes are sensitive to the substrate topographic features, as well as the substrate stiffness. Photolithography has been widely applied to generate topographic features on a variety of biomaterial substrates, with the aim of enhancing cardiomyocyte alignment.^71–76^ As two examples of commonly used biomaterials, PEG- and PDMS-based substrates can be processed to form grooved structures at the micro- and nanoscale. The cardiomyocytes cultured on these grooved substrates showed enhanced structural alignment and improved Ca^2+^ cycling.^77–79^ A polystyrene substrate was fabricated with nanopillars of different sizes to create a gradient of nanoscopic topography. The gradient nanopatterns increased the expression of integrin-α5, vinculin, and cofilin of mesodermal precursor cells through the inhibition of cytoskeleton disassembly and further enhanced cell spreading and cardiac differentiation.^80^

A functioning heart constantly experiences mechanical stretching during filling (preload) and contraction against systemic vascular resistance during ejection (afterload). The investigation of cardiac load (preload and afterload) on the maturation of cardiomyocytes is largely dependent on *in vitro* tissue models. Previous studies based on neonatal rat cardiomyocytes and embryonic chick cardiomyocytes showed that cyclic stretching could increase sarcomeric α-actinin expression, induce sarcomere growth, and enhance contractile forces.^81–83^ Furthermore, mechanical stretching also increased the expression of proteins on the intercalated disks, such as connexin 43 (Cx43), plakoglobin, desmoplakin, and *N*-cadherin, indicating an enhancement of cell–cell communications.^84^

More recently, mechanical load was explored as a critical factor in cardiac disease modeling. In one study, cardiac microtissues were generated by assembling hiPSC-CMs within microfabricated dogbone structures on fibronectin-grafted PDMS-based substrates with different stiffnesses. Cardiac microtissues experiencing high substrate stiffness showed an increase in contractility after 9 days of culture, followed by a decrease in contractility at day 16; this suggested that the high cardiac afterload generated by the stiff substrate might lead to hypertrophic remodeling of the myocardium.^85^ In the area of disease modeling, a pioneering study showed that the twitch force difference between wild type and titin-mutated hiPSC-derived cardiac microtissues was increased from 1.93 *μ*N to 4.40 *μ*N, along with an increase in PDMS pillar stiffness. This result indicates that the mechanical overload from high pillar stiffness could deteriorate the contractile deficits of hiPSC-CMs with genetic defects.^86^ A similar study suggested that genetic defects and mechanical overload synergistically induced severe contractile deficits for cardiac microtissues grown on filamentous matrices with different mechanical properties. Compared with wild-type cardiac microtissues, the deficiency in contraction force of microtissues with the MYBPC3^−/−^ mutation only appeared when the tissues experienced a high mechanical load.^87^ Recently, hiPSC-CMs with homozygous cardiovascular-risk alleles showed asynchronous contraction only when the cells were cultured on a hyaluronic acid hydrogel substrate with high stiffness.^88^ These studies collectively indicate that the mechanical overload could induce severe pathological phenotypes in hiPSC-CMs with genetic defects, highlighting the importance of environmental factors in disease initiation and progression.

Since the main function of the myocardium is to power blood flow, it is critical to understand how dynamic shear flow affects cardiac cell phenotypes. Shear stress generated by using a perfusion system with a laminar flow chamber and a flow regulator was found to trigger the recruitment of potassium channels in atrial myocytes.^89^ Moreover, shear stress was also found to promote stem cell differentiation toward the cardiac lineage by increasing the expression of cardiac genes, elevating the secretion of atrial natriuretic peptide (ANP),^90,91^ and stimulating local Ca^2+^ release.^74^ External compression also serves as an important mechanical signal since direct cardiac compression could decrease the preload volume of heart ventricles^92^ and enhance ventricular contractile efficiency during ejections.^93,94^ A bioreactor was designed to apply compressive force by manipulating the downward motion of a piston, and it was found that cardiomyocytes subject to intermittent compression showed better structural alignment and higher expression of cardiac markers; this might be attributed to the increased expression of bFGF, transforming growth factor (TGF-β), and Cx43.^95^

### B. Dynamic cell mechanobiology

Compared with external stimulations or passive materials, stimuli-responsive materials can undergo gradual and reversible changes in their mechanical properties in response to environmental elements. If stimuli-responsive materials are used as cell culture systems, the cells can access dynamic mechanical cues; studies of this type can further advance the field of dynamic cell mechanobiology. For instance, a shape memory polymer (SMP)-based substrate was fabricated using polycaprolactone as the base polymer and allyl alcohol as the plasticizer. The dynamic microgrooves formed on this substrate could deliver mechanical cues to the human MSCs to induce their elongation and differentiation during shape recovery.^96^ A PEG-based dynamic hydrogel with the ability of stiff-to-soft transition was also used to direct mechanobiological changes of MSCs. Human MSCs on stiff hydrogels were prone to adipogenic differentiation in a short culture period, and they underwent osteogenic differentiation when the culture period was extended. Interestingly, a long-term culture on a stiff hydrogel provided a sufficient dose of mechanical signals to trigger YAP/TAZ activation in MSCs, causing an irreversible change in cell phenotype.^97^

In the native myocardium, the ECM not only provides a structural support to the cardiac cells but also plays a pivotal role in regulating cardiac development during early embryogenesis. Therefore, reproduction of the spatiotemporal characteristics of the dynamic ECM would help us gain a better understanding of cardiac mechanobiology during myocardial development, which can be used to formulate new approaches to enhance hiPSC-CM maturation *in vitro*. Taking advantage of stimuli-responsive biomaterials, we are able to study cell remodeling and phenotype conversion due to progressive changes in the extracellular microenvironment. In particular, the programable mechanical properties of stimuli-responsive biomaterials provide us with great opportunities to study dynamic cardiac mechanobiology during cardiac development and disease progression.

### C. Dynamic substrate topography on cardiac mechanobiology

**FIG. 3.**
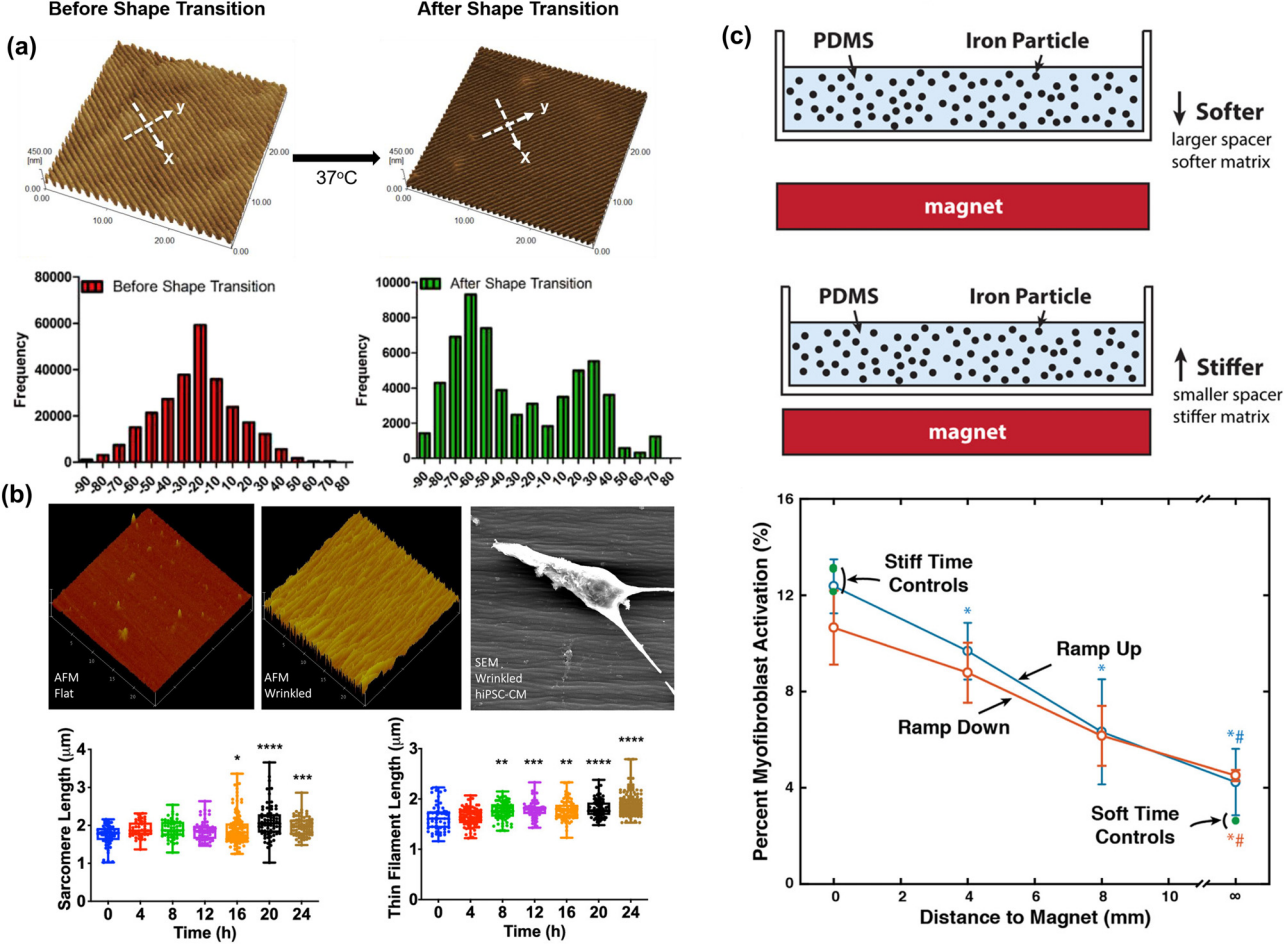
Stimuli-responsive biomaterials for cardiac dynamic mechanobiology. (a) Polycaprolactone-based thermo-responsive SMP enabled a dynamic topography with an orthogonal direction change in response to thermal stimulation. Anisotropic cardiomyocyte focal adhesions became isotropically oriented in response to a shape transition.^100^ Reprinted with permission from Mengsteab *et al.*, “Spatiotemporal control of cardiac anisotropy using dynamic nanotopographic cues,” Biomaterials **86**, 1–10 (2016). Copyright 2016 Elsevier. (b) Dynamic topography was also achieved on a tBA-*co*-BA SMP-based substrate, which could transition from a flat surface to a wrinkled surface, as shown in the atomic force micrographs. This dynamic flat-to-wrinkled transition could mediate cardiomyocyte alignment and remodeling of cardiac myofibrils.^101^ Reprinted with permission from Sun *et al.*, “Progressive myofibril reorganization of human cardiomyocytes on a dynamic nanotopographic substrate,” ACS Appl. Mater. Interfaces **12**(19), 21450 (2020). Copyright 2020 American Chemical Society. (c) The stiffness of a magneto-responsive substrate can be reversibly changed by adjusting the distance between the magnet and a PDMS substrate incorporated with ironic particles. This two-way stiffness change could reversibly modulate the myofibroblast activation of cardiac fibroblasts.^70^ Reprinted with permission from Corbin *et al.*, “Tunable and reversible substrate stiffness reveals a dynamic mechanosensitivity of cardiomyocytes,” ACS Appl. Mater. Interfaces **11**(23) 20603 (2019). Copyright 2019 American Chemical Society.

Shape memory polymers enable the fabrication of cell culture substrates with dynamic topographies, owing to their ability of strain recovery under specific stimuli.^98,99^ Polycaprolactone was utilized to fabricate a thermo-responsive SMP with dynamic nanogrooves. The direction of the nanogrooves could be changed orthogonally by switching from a temporary surface pattern to a permanent surface pattern, triggered by a temperature increase. During this process, anisotropic nuclei orientation and contraction directions became isotropic, accompanied by a decrease in cardiomyocyte elongation. Moreover, focal adhesions in parallel with the nanogrooves became randomly distributed in response to the topographic reorganization of this dynamic substrate [Fig. 3(a)].^100^ In another study, poly(tert-butyl acrylate-*co*-butyl acrylate) (*t*BA-*co*-BA) was used to fabricate an SMP with a low glass transition temperature under hydrated conditions. With a polyelectrolyte multilayer (PEM) coating, this SMP-PEM composite substrate enabled a flat-to-wrinkled topographical transformation in response to a temperature increase from 30 °C to 37 °C. The hiPSC-CMs grown on this SMP-PEM substrate showed enhanced cell alignment at 36 h of culture after the initiation of wrinkle formation. The thin filament length was found to increase after 8 h, while the distance between two adjacent Z-disks was found to increase after 16 h. The sequential reorganization of myofibrillar subunits indicated that the early assembly of actin filaments provided a stable base to recruit α-actinin to form Z-bodies during myofibrillogenesis [Fig. 3(b)]. Moreover, the length of focal adhesions on hiPSC-CMs decreased 4 h after wrinkle formation, indicating that focal adhesion rearrangement preceded myofibrillar remodeling and morphological changes of cardiomyocytes.^101^

### D. Effect of dynamic substrate stiffness on cardiac mechanobiology

The stiffness of functioning adult myocardium is 10–15 kPa,^102,103^ while the stiffness of mesoderm, where early cardiac development originates, is only approximately 500 Pa.^104^ This dramatic stiffening of ECM during the tissue morphogenesis process plays a critical role in the regulation of cell differentiation. To mimic this stiffening process, substrates with dynamic tunable stiffness were used to investigate how cell–matrix interactions affect developmental cardiac mechanobiology. Hyaluronic acid hydrogel showed time-dependent post-polymerization stiffening from 2 kPa to 8 kPa, which mimicked cardiomyocyte maturation from the softer mesoderm germ layer to stiffer adult myocardium. Chicken embryonic heart cells grown on this gradual stiffening hyaluronic acid hydrogel showed better myofibril alignment and stronger TNNT2 expression than cells grown on polyacrylamide hydrogel with static stiffness.^105^

The stiffness of a photosensitive PDMS substrate can be increased by ultraviolet curing, enabling the fabrication of a dynamic soft-to-stiff substrate. After stiffening of the substrate, cardiac fibroblasts transformed into myofibroblasts with a high expression of α-smooth muscle actin.^106^ In a recent study, a PDMS-based substrate with dynamic stiffness was fabricated by mixing it with iron particles, which were subjected to a magnetic field. To investigate the cellular responses to the dynamic stiffness, this magneto-responsive system was used in two groups: a dynamic stiff-to-soft group and a dynamic soft-to-stiff group. The stiff-to-soft transition could reverse both cell spreading and myofibroblast activation induced by the previous substrate stiffening process. Interestingly, gene expressions of hiPSC-CMs in these two dynamic groups showed different trends. For instance, MYH6 expression was negatively correlated with stiffness, while MYH7 expression increased in both stiff-to-soft and soft-to-stiff groups. These results suggest that there are different cardiac gene expression patterns in the dynamic mechanobiological remodeling of cardiomyocytes in response to two-way dynamic stiffness [Fig. 3(c)].^70^

## IV. CONCLUSION AND FUTURE PERSPECTIVE

Stimuli-responsive biomaterials are attractive carriers for delivering stem cells and therapeutic drugs for heart disease treatment. Numerous animal studies have demonstrated the functionality of stimuli-responsive biomaterials in enhancing local cell or drug retention at the injured heart to promote cardiac regeneration. By designing and modulating the molecular composition and structure, new smart active biomaterials could have refined responsiveness and controlled release profiles in concordance with the rate of native heart regeneration. However, stimuli-responsive biomaterials are still at an early age of development, with plenty of challenges in engineering and characterization methods. Currently, the property change of smart materials is primarily triggered by general environmental stimuli in the diseased heart, while responsiveness to tissue- or cell-specific stimuli could escalate the localization accuracy of therapeutic components. Conversely, the lack of *in vivo* characterization techniques in real time hinders progression in uncovering regeneration mechanisms and *in situ* comparison between different delivery systems. Although it will be a long journey to achieve the rational design and standardization of these smart carriers, new stimuli-responsive biomaterials will be continuously explored for various applications in cardiac tissue engineering and regenerative medicine.

There should be a new focus on the dynamic electrical microenvironment, to expand our knowledge of cardiac responses to extracellular changes. For example, an electroresponsive material, poly(pyrrole) (PPy), was incorporated into acid-modified silk fibroin to fabricate an electroresponsive polymer substrate.^107^ Cardiomyocytes grown on this substrate showed an increase in the sarcomere length and the Z-bandwidth, with an upregulated expression of the gap junction and cardiac-specific genes. In recent years, carbon nanotubes (CNTs) have been extensively explored as conductive materials for cardiac tissue engineering applications. Embedding CNTs to enhance the conductivity of different polymer scaffolds has been shown to improve intercellular communication and cardiomyocyte maturation.^107–111^ In the future, the utility of electrical-responsive biomaterials will be a promising approach to dynamically manipulate the electroconductive microenvironment, which offers great opportunities to study the signaling pathways of CMs related to electrical stimulation and transduction.

In the field of cardiac mechanobiology, stimuli-responsive biomaterials enable the development of new dynamic substrates that have been proved to induce cardiomyogenesis and promote cardiomyocyte maturation. The manipulation of biomaterial properties provides novel approaches to mimic the dynamic changes of cardiac microenvironments during early heart development and disease progression, making it possible to investigate the molecular mechanisms underlying cardiac responses to these environmental factors. In addition, the viscoelasticity of the ECM is directly related to the formation of a focal adhesion complex and the transduction of a mechanical load, which are critical for the normal contractile functions of cardiomyocytes. Further understanding of biological responses to dynamic viscoelasticity would significantly enrich our knowledge of dynamic cardiac mechanobiology. Most stimuli-responsive biomaterials used for dynamic cardiac mechanobiology are still primarily two-dimensional systems with tunable topographies, stiffness, and so on. Future research is moving toward the establishment of three-dimensional systems, which can provide cells with a microenvironment exhibiting a higher similarity to native conditions.

## AUTHORS' CONTRIBUTIONS

H.S. and C.W. contributed equally to this work.

## Data Availability

The data that support the findings of this study are available from the corresponding author upon reasonable request.
